# Perceived mental health, work, and life stress in association with the amount of weekly alcohol consumption among Canadian adults who have ever drank

**DOI:** 10.1186/s12889-022-14240-3

**Published:** 2022-10-05

**Authors:** Nigatu Geda, Cindy Feng

**Affiliations:** 1 Center for Population Studies, College of Development Studies, Addis Ababa University, Addis Ababa, Ethiopia; 2 Department of Community Health and Epidemiology, Faculty of Medicine, Dalhousie University, Nova Scotia, Canada

**Keywords:** Alcohol, Mental health, Life stress, Work stress

## Abstract

**Background:**

Excess alcohol consumption has multifaceted adverse impacts at individual, household, and community levels. The study primarily aims at assessing the role of perceived health and stress in alcohol consumption among adults in Canada who have ever drank.

**Methods:**

The study was conducted based on a total of 35,928 Canadian adults aged 18 and above who have ever drank, extracted from the 2017–2018 Canadian Community Health Survey (CCHS) data. A mixed-effect Negative Binomial (NB) regression model was used to determine the effects of three key risk factors (perceived mental health, life stress, and work stress) in association with the self-reported number of weekly alcohol consumption, controlling for other variables in the model.

**Results:**

The study found that regular alcohol consumption among ever drank Canadian adults is high, with the self-reported number of weekly alcohol consumption ranging from 0 to 210. The results of adjusted mixed-effect NB regression showed that the expected mean of alcohol consumption was significantly higher among those with a poorer perception of mental health, higher perceived work, and life stress. Nonsmokers have a much lower mean score of alcohol consumption compared to those who smoke daily. There was a significant interaction between racial background and the three key predictors (perceived mental health, life stress, and work stress).

**Conclusion:**

Given the reported perceived health and stress significantly impacts alcohol consumption, the findings suggested improving individual/group counseling, and health education focusing on home and work environment to prevent and manage life stressors and drivers to make significant program impacts.

## Background

Excess alcohol intake is a growing public health concern around the globe as it has multifaceted adverse impacts at individual, household, and community levels. Some of the direct health implications of alcohol misuse include dependency, organ damage, diabetes, cardiovascular disease, liver cirrhosis, and various types of cancer [1–3]. The social impacts include its adverse influence on the entire family’s functioning, self-inflicted injuries, disruptions in social relationships, and others [4]. Worldwide, alcohol abuse accounted for approximately 3.3 million deaths and 139 million disability-adjusted life years due to injury and morbidity [5]. In Canada, excess alcohol intake among the adult population has been a growing concern as it is increasingly impacting the health and safety of individuals, families, and communities. According to the 2018 study conducted by the Canadian Centre on Substance Use and Addiction, the economic cost of alcohol consumption was estimated as $15 billion per year in terms of lost productivity, justice costs, and more [6].

Previous studies around the world reported that a range of socio-economic factors contributes to individuals’ drinking practices [7, 8]. Socio-economic status (SES), including income, occupation, educational attainment, social position, and other variables, has been generally linked to the risk of excessive alcohol intake [9–11]. Studies in Canada found a range of variables affecting alcohol consumption. For example, a study based on 2009/2010 Canadian Community Health Survey (CCHS) data indicated significant differentials in alcohol consumption by gender, marital status, residence, cigarette smoking, income, education, and perceived health status [9]. The Public Health Agency of Canada reported a range of factors influencing high alcohol consumption, including alcohol access and affordability, gender norms, social environment “(e.g., social and cultural practices, loss of cultural identity, stigma, social networks, and supports),” socio-economic position, occupation type, individual motivations for drinking, coping abilities and lack of knowledge about the risks [12].

Of the important psychosocial factors affecting alcohol consumption, perceived stress (both life and work-related stress) and mental health are becoming important concerns in fast-developing urban life. Though the connection between stress and alcohol consumption was reported in early alcohol research, recent epidemiological data also established an increased stress level is associated with excess alcohol use. In the tension-reduction hypothesis, stress was seen to increase anxiety, and in response, alcohol was consumed to reduce the anxiety [13]. Excess alcohol consumption decisions may also be impacted by underlying mental health concerns [14]. In its most recent survey, the Public Health Agency of Canada reported that excess alcohol consumption was much higher for those who had lower self-perceived mental health during the COVID-19 pandemic [15]. Lower self-perceived health status among Canadian adults was also associated with increased odds of risky infrequent or frequent drinking practices [9]. The association between psychosocial factors and alcohol intake may also differ according to the individuals’ socio-demographic status [15], which has not been adequately explored in a Canadian context.

The very few studies conducted in Canada on the subject had both conceptual and methodological limitations. Some of them used the frequency of alcohol intake during the last 12 months as an outcome variable which is commonly impacted by memory lapse and misreporting [15, 16]. Also, none of the studies employed multilevel modeling to account for variations at the cluster level [15]. In most instances, previous studies addressed only selected socio-economic risk factors, and little or no attention was given to perceived health and stress dimensions. Also, there has been little attempt to explore the interaction between main exposure variables and certain social demographic and economic risk factors with alcohol consumption. Studying the risk factors (more importantly, their interaction effect) of excess alcohol consumption may provide insight into the key determinants of excess alcohol consumption and help guide relevant and informed alcohol-related policies. The main objective of the present study, thus, is to examine the role of perceived stress and mental health on alcohol consumption among adults (age18 and above) in Canada who have ever drank.

## Methods

### Data source, study design and study population

The study is based on data from the 2017–2018 Canadian Community Health Survey (CCHS), which is a multistage complex cross-sectional survey that collected socio-economic and health information from 113,290 participants aged 12 + drawn from all Canadian provinces and territories [17]. For the present analysis, a total of 35,928 ever drinkers aged 18 and above were extracted from a total of 104,636 respondents. The CCHS follows a standard protocol and procedures of data collection.

### Study variables

#### Dependent variable

The dependent variable of the study is weekly alcohol consumption (WAC), measured by asking respondents how many drinks they had every day on average during the last seven days prior to the survey date? Responses were provided for each day, and then converted to WAC.

#### Primary explanatory variables of interest

The main explanatory variables in this study were the level of life and work stresses and perceived mental health measures. They were all self-reported exposures. Participants’ responses on the two stress variables were coded into five categories: not at all stressful, not very stressful, a bit stressful, quite a bit stressful, and extremely stressful. Perceived mental health was coded as poor, fair, good, very good and excellent. Data on both life and work stress variables were collected based on respondents’ experiences of stress during the 12 months preceding the survey. The question about life stress was `thinking about the amount of stress in your life, what would you say most days are?’. Similarly, the work stress question was asked as ‘what would you say most days at work were?’

#### Other explanatory variables

The analysis included a number of control variables such as age, sex, household income, ethnicity, educational status, marital status, immigration status and household food security. Age was classified into five groups: <=24, 25–34, 35–50, 51–64 and 65 + years old. Ethnicity was grouped as white, aboriginal, or visible minority. Respondents’ educational status was categorized into less than secondary school graduation, secondary school graduation, and post-secondary certificate diploma/university education. Marital status was defined as: married, common-law, widowed/divorced/separated, and single/never married. Respondents were grouped into five household income categories, with $20,000 increments beginning with zero. It was categorized as no income or less than $20,000, $20,000 to $39,999, $40,000 to $59,999, $60,000 to $79,999, and $80,000 or more per year. Household food access was categorized into food secured, moderately food insecure, and severely food insecure. Current smoking cigarettes was coded as daily, occasionally, and not at all.

### Statistical analyses

CCHS 2017–2018 was collected over 97 health regions across Canada, so the responses of the individuals from the same health region (cluster) tend to be similar due to the underlying factors at the level of health region. As a result, mixed-effect Poisson, and mixed-effect Negative Binomial (NB) models are considered in this study. The inclusion of a random effect term for health region in the model addresses the correlation among the observations from the same health region. Multicollinearity among the explanatory variables was checked using the Variance Inflation Factor (VIF), with VIFs > 2.5 indicating multicollinearity problems [18]. Bivariate mixed-effect regression models were firstly run to examine the associations between covariates and the outcome variable. As a rule of thumb, potential variables with a p-value < 0.20 were further tested in the multivariable mixed-effect models [18]. Alkaline Information Criteria (AIC) was used to compare the model fits of mixed-effect Poisson and mixed-effect NB models. In cases where respondents have missed reporting on any of the questions asked, we used the commonly used technique of listwise deletion, which deletes the cases containing missing data in the variables that are relevant to the analysis being carried out. All analyses were weighted using CCHS’s prescribed weight variable [17, 19], accounting for the complex survey design. SPSS version 26 was used to carry out the analysis.

## Results

The weekly consumption of alcohol (number of bottles of alcohol they had during the last seven days prior to the survey date) is a count variable ranging from 0 to 210 (Median = 2.0, IQR = 6) based on the 35,928 respondents who reported having drunk alcohol ever. Table 1 presents the percentage distribution of respondents by selected characteristics. Male respondents account for about half of the respondents. In terms of racial background, close to three fourth of the study participants were white Canadians, and 70% of the respondents were non-immigrants (Canadian-born). A little more than half of the respondents were married, and about a quarter of them was single. A nearly equal proportion of respondents are in the age group 35–49 years (25%), and 50–64 -years age group (26%). More than half of the respondents are from households with an annual income of $80,000 or more. A significant proportion of respondents reported that they were not smoking during the survey period. With regards to perceived mental health, 38% and 31% of the study participants reported ‘very good’ and ‘excellent’. Close to two-thirds of the respondents reported experiencing moderate (a bit stressful) to extreme life stress (see Table 1). Similarly, a little more than two-thirds of the study participants reported moderate (bit stressful) to extreme work-related stress.

**Table 1:** Distribution of the characteristics of participants, CCHS 2017-18, Canada. (n = 35,928)

Background Characteristics	Weighted %	Missing (%)
**Sex**		0.0
Male	51.1	
Female	48.9	
**Ethnicity**		0.0
White	71.1	
Aboriginal	3.1	
Visible minority	25.8	
**Age**		0.0
18–34	30.4	
35–49	25.2	
50–64	26.2	
65 and above	18.1	
**Marital status**		0.2
Married	52.6	
Common-law	9.6	
Widowed/Divorced/Separated	11.7	
Single	26.1	
**Educational level**		1.5
Less than secondary school graduation	7.6	
Secondary school graduation	25.3	
Post-secondary certificate diploma/university	67.2	
**Immigration Status**		2.0
Landed immigrant	30.1	
Non-immigrant (Canadian born)	69.9	
**Household food security**		2.4
Food secure	92.7	
Moderately food insecure	4.6	
Severely food insecure	2.6	
**Household income**		0.0
No income or less than $20,000	5.3	
$20,000 to $39,999	10.4	
$40,000 to $59,999	13.0	
$60,000 to $79,999	12.4	
$80,000 or more	59.0	
**Smoking**		0.0
Daily	12.1	
Occasionally	4.9	
Not at all	83.0	
**Perceived mental health**		2.6
Poor	1.8	
Fair	6.2	
Good	22.9	
Very good	38.3	
Excellent	30.8	
**Life stress**		0.3
Not at all stressful	11.2	
Not very stressful	24.1	
A bit stressful	42.3	
Quite a bit stressful	19.0	
Extremely stressful	3.4	
**Work stress level** *		1.3
Not at all stressful	8.2	
Not very stressful	20.5	
A bit stressful	43.6	
Quite a bit stressful	22.8	
Extremely stressful	4.9	

*Respondents who reported not working (n = 12,560) were removed

A bivariate mixed-effect NB is presented in Table 2 to select potential variables for the multivariable NB regression analysis. As a rule of thumb, variables with p-value < 0.2 will be further entered into the multivariable NB model. All the variables are significantly associated with WAC (p-value < 0.001). The responses of those who had no work during the reference period were marked as ‘not applicable (n = 12,560) and were excluded from the final multivariable regression analysis. In addition, 1285 respondents were removed due to missing values of other explanatory variables. Therefore, the sample size of the final multivariable mixed-effect NB model was 22,083.

**Table 2:** Unadjusted Risk Ratio (RR) with 95% confidence intervals (CI) for the bivariate analysis of main exposure variables and socio-demographic characteristics in association with WAC, CCHS, 2017–2018, Canada

	Unadjusted RR (95% CI)	p-values
**Socio-demographic variables**		
**Sex**		
Male	1	**0.000**
Female	**0.52(0.52–0.52)**	**0.000**
**Ethnicity**		
White	1	
Aboriginal	**1.12(1.12–1.13)**	**0.000**
Visible minority	**0.44(0.44–0.44)**	**0.000**
**Age**		
18–34	1	
35–49	**1.09(1.08–1.09)**	**0.000**
50–64	**1.18(1.18–1.19)**	**0.000**
65 and above	**0.95(0.95–0.95)**	**0.000**
**Marital status**		
Married	1	
Common-law	**1.48(1.47–1.48)**	**0.000**
Widowed/Divorced/Separated	**0.93(0.93–0.93)**	**0.000**
Single	**1.05(1.04–1.05)**	**0.000**
**Educational level**		
Less than secondary school graduation	1	
Secondary school graduation	**1.04(1.03–1.04)**	**0.000**
Post-secondary certificate diploma/university	**0.96(0.95–0.96)**	**0.000**
**Immigration Status**		
Landed immigrant	1	
Non-immigrant (Canadian born)	**1.87(1.86–1.87)**	**0.000**
**Household food security**		
Food secure	1	
Moderately food insecure	**0.85(0.84–0.85)**	**0.000**
Severely food insecure	**0.90(0.90–0.91)**	**0.113**
**Household income**		
No income or less than $20,000	1	
$20,000 to $39,999	**0.91(0.91–0.92)**	**0.000**
$40,000 to $59,999	**1.06(1.06–1.07)**	**0.000**
$60,000 to $79,999	**1.07(1.07–1.08)**	**0.000**
$80,000 or more	**1.30(1.30–1.31)**	**0.000**
**Smoking**		
Not at all	1	
Occasionally	**1.76(1.75–1.77)**	**0.000**
Daily	**1.92(1.91–1.93)**	**0.000**
**Main exposure variables**		
**Life stress**		
Not at all stressful	**1**	
Not very stressful	**1.05(1.04–1.05)**	**0.000**
A bit stressful	**0.93(0.93–0.93)**	**0.000**
Quite a bit stressful	**1.11(1.11–1.12)**	**0.000**
Extremely stressful	**1.45(1.47–1.46)**	**0.000**
**Work stress level**		
Not at all stressful	**1**	
Not very stressful	**1.07(1.07–1.08)**	**0.000**
A bit stressful	**1.14(1.14–1.15)**	**0.000**
Quite a bit stressful	**1.13(1.13–1.14)**	**0.000**
Extremely stressful	**1.74(1.73–1.76)**	**0.000**
**Perceived mental health**		
Poor	1	
Fair	**0.86(0.85–0.86)**	**0.000**
Good	**0.81(0.80–0.81)**	**0.000**
Very good	**0.80(0.79–0.80)**	**0.000**
Excellent	**0.77(0.77–0.78)**	**0.000**

The model comparison indicated mixed effect NB model provided a much better fit to the data (AIC = 29,331,683) compared to the mixed effect Poisson model (AIC = 74,307,097). As a result, we present the results based on the mixed-effect NB model.

**Table 3:** Multivariable mixed-effect NB regression for examining the association between socio-demographic characteristics with the WAC, CCHS, 2017–2018, Canada. (n = 22,083)

	Adjusted RR (95% CI)	p-values
**Socio-demographic variables**		
**Sex**		
Male	1	**-**
Female	**0.56(0.55–0.56)**	**0.000**
**Age**		
18–34	1	**-**
35–49	**1.07(1.07–1.08)**	**0.000**
50–64	**1.17(1.16–1.17)**	**0.000**
65 and above	**1.14 (1.13–1.15)**	**0.000**
**Marital status**		
Married	1	**-**
Common-law	**1.25(1.25–1.26)**	**0.000**
Widowed/Divorced/Separated	**1.01(1.01–1.02)**	**0.000**
Single	**1.10(1.10–1.11)**	**0.000**
**Educational level**		
Less than secondary school graduation	1	**-**
Secondary school graduation	**1.01(1.00-1.01)**	**0.008**
Post-secondary certificate diploma/university	**1.06 (1.05–1.06)**	**0.000**
**Immigration Status**		
Landed immigrant	1	**-**
Non-immigrant (Canadian born)	**1.37(1.36–1.37)**	**0.000**
**Household food security**		
Food secure	1	**-**
Moderately food insecure	**0.83(0.83–0.84)**	**0.000**
Severely food insecure	**0.77(0.76–0.77)**	**0.000**
**Household income**		
No income or less than $20,000	1	**-**
$20,000 to $39,999	**0.93(0.92–0.93)**	**0.000**
$40,000 to $59,999	**1.07(1.07–1.08)**	**0.000**
$60,000 to $79,999	**1.06(1.06–1.07)**	**0.000**
$80,000 or more	**1.19(1.18–1.20)**	**0.000**
**Smoking**		
Not at all	1	**-**
Occasionally	**1.91(1.89–1.92)**	**0.000**
Daily	**1.86(1.85–1.86)**	**0.000**

**Fig. 1:**
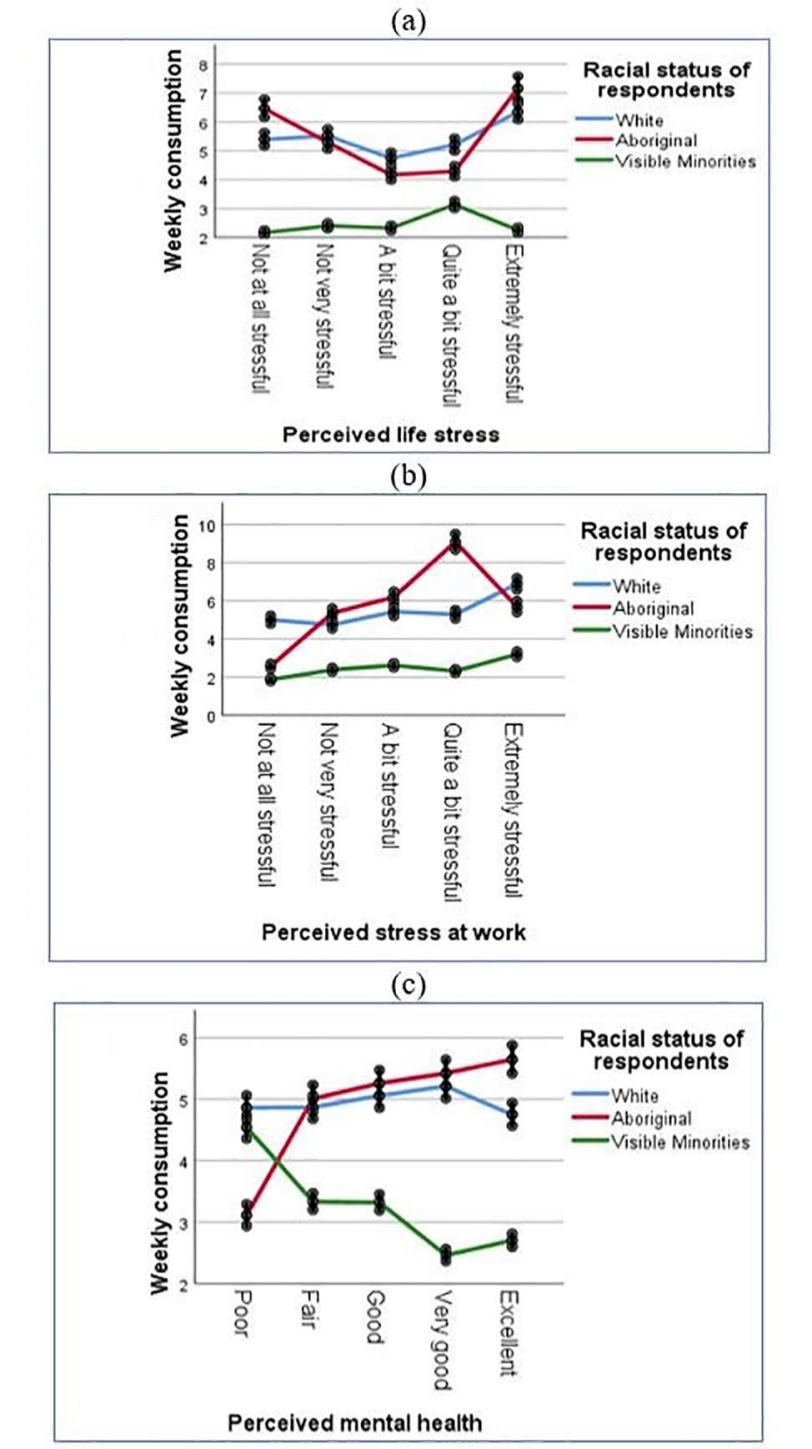
Estimated marginal means of WAC for the significant interactions between (a) perceived life stress and racial status (b) perceived work stress and racial status and (c) perceived mental health and racial status

Table 3 presents the multivariable mixed effect NB regression for examining the association between socio-demographic characteristics with WAC. Significant interactive effects were identified between the main exposure variables and the WAC, with the results presented in Tables 4, 5 and 6; Fig. 1. The variance component of the random effect term is estimated as 6.17(95% CI: 5.92–6.44) (p-value < 0.001), indicating that there was significant variation in WAC among the health regions. The expected mean WAC was significantly lower among females compared to males (RR = 0.56, 95% CI: 0.55–0.56). The expected mean WAC was significantly higher for older age groups compared to the age group 18–34. Compared to married respondents, the mean WAC was significantly higher for common-law, widowed/divorced/separated, and single. Those who completed high school and had post-secondary education had higher mean WAC (RR = 1.00; 95% CI: 1.00-1.01 and RR = 1.06; 95% CI: 1.05–1.06, respectively). The expected mean WAC was also significantly higher among non-immigrants (RR = 1.37, 95% CI: 1.36–1.37) than immigrants. The result indicates that the risk of WAC was higher among higher-income groups. Conversely, those living in food-insecure households had a significantly lower WAC. The mean WAC is 1.91 times higher among daily smokers compared to those who never smoked (RR = 1.91; 95% CI: 1.90–1.92). Similarly, the WAC was 1.86 times higher among occasional smokers (RR = 1.86; 95% CI:1.85–1.86).

Three statistically significant interactions emerged between perceived life stress and racial status of respondents (p-value < 0.001), perceived work stress and racial status of respondents (p-value < 0.001), and perceived mental health and racial status of respondents (p-value < 0.001). The adjusted marginal means of WAC and corresponding 95% confidence intervals (CI) stratified by the significant interaction terms are presented in Tables 4, 5 and 6, respectively, which are also depicted in Fig. 1.

**Table 4:** Adjusted marginal means of WAC and corresponding 95% confidence intervals (CI) for the significant interaction between respondents’ perceived life stress and racial status

Perceived life stress	Racial status	Estimate		95% CI	
		**Mean**	**Std. Error**	**Lower**	**Upper**
Extremely stressful	Visible Minorities	2.23	0.05	2.16	2.36
	Aboriginal	7.16	0.21	6.76	7.59
	White	6.35	0.14	6.08	6.63
Quite a bit stressful	Visible Minorities	3.14	0.07	3.01	3.28
	Aboriginal	4.29	0.09	4.10	4.49
	White	5.21	0.11	4.99	5.44
A bit stressful	Visible Minorities	2.33	0.05	2.23	2.43
	Aboriginal	4.17	0.09	3.99	4.36
	White	4.75	0.10	4.55	4.95
Not very stressful	Visible Minorities	2.41	0.05	2.30	2.51
	Aboriginal	5.29	0.12	5.06	5.54
	White	5.52	0.12	5.29	5.76
Not at all stressful	Visible Minorities	2.17	0.05	2.08	2.26
	Aboriginal	6.47	0.16	6.16	6.79
	White	5.40	0.12	5.17	5.63

**Table 5:** Adjusted marginal means of WAC and corresponding 95% confidence intervals (CI) for the significant interaction between respondents’ perceived stress at work and racial status

		Estimate			95% CI		
**Perceived stress at work**	**Racial status**	**Mean**		**Std. Error**	**Lower**	**Upper**	
Extremely stressful	Visible Minorities		3.19	0.07	3.06	3.34	
	Aboriginal		5.69	0.15	5.41	5.99	
	White		6.88	0.15	6.59	7.18	
Quite a bit stressful	Visible Minorities		2.31	0.05	2.22	2.42	
	Aboriginal		9.07	0.21	8.67	9.49	
	White		5.28	0.12	5.06	5.52	
A bit stressful	Visible Minorities		2.62	0.06	2.51	2.73	
	Aboriginal		6.19	0.14	5.92	6.47	
	White		5.42	0.12	5.20	5.66	
Not very stressful	Visible Minorities		2.39	0.05	2.29	2.49	
	Aboriginal		5.35	0.12	5.11	5.60	
	White		4.74	0.10	4.54	4.95	
Not at all stressful	Visible Minorities		1.87	0.04	1.79	1.95	
	Aboriginal		2.57	0.06	2.45	2.70	
	White		5.00	0.11	4.79	5.22	

**Table 6:** Adjusted marginal means of WAC and corresponding 95% confidence intervals (CI) for the significant interaction between respondents’ perceived mental health and racial status

		Estimate			95% CI		
**Perceived mental health**	**Racial status**	**Mean**		**Std. Error**	**Lower**	**Upper**	
Excellent	Visible Minorities		2.70	0.05	2.60	2.81	
	Aboriginal		5.64	0.12	5.42	5.88	
	White		4.75	0.10	4.57	4.94	
Very good	Visible Minorities		2.46	0.05	2.37	2.56	
	Aboriginal		5.42	0.11	5.21	5.64	
	White		5.21	0.10	5.01	5.42	
Good	Visible Minorities		3.32	0.07	3.19	3.45	
	Aboriginal		5.26	0.11	5.05	5.47	
	White		5.06	0.10	4.86	5.26	
Fair	Visible Minorities		3.33	0.07	3.20	3.47	
	Aboriginal		5.01	0.11	4.80	5.23	
	White		4.87	0.10	4.68	5.06	
Poor	Visible Minorities		4.54	0.10	4.36	4.73	
	Aboriginal		3.11	0.09	2.94	3.29	
	White		4.86	0.10	4.67	5.06	

Table 4 and Fig. 1 (a) indicate that the expected mean WAC among the visible minorities slightly increased as the perceived life stress increased and reached the peak for those who reported quite a bit stressful and then declined for those who reported extreme stress. The expected mean of the WAC among the aboriginals and whites was significantly higher than the visible minority, exhibiting a U-shaped relationship with the perceived life stress level. In particular, the WAC was considerably higher for the aboriginal people than the white and visible minorities for those who reported not at all stressful (expected mean = 6.47, 95% CI: 6.16–6.79) or extremely stressful (expected mean = 7.16, 95% CI:6.76–7.59). Table 5; Fig. 1(b) reveal that the expected mean WAC increased sharply among the aboriginal people as the perceived work stress level increased and peaked at “quite a bit stressful”. The percentages reported in Table 5 indicated that the expected mean WAC also gradually increased as the work stress level increased among the whites and visible minorities, with the expected mean WAC level among the whites being much higher than the visible minorities. For example, at extreme work stress situations, whites had an expected mean WAC of 6.88 (95% CI:6.59–7.18). Aboriginals had much higher mean WAC at moderate (expected mean = 6.19, 95% CI: 5.92–6.47) and a higher level of stress levels (expected mean = 9.07, 95% CI:8.67–9.49). Table 6; Fig. 1(c) indicate that when the perceived mental health was poor, the WAC among the visible minority was also considerably high and comparable to whites, followed by a sharp decline at a better perceived mental health. Conversely, the WAC was significantly lower among the aboriginals for those with poor mental health and higher for those with better mental health. WAC was consistently higher among whites than visible minorities regardless of their mental health status and slightly lower than the aboriginals when the perceived mental health is fair to excellent.

## Discussion

Our study found that WAC was significantly higher for respondents reporting moderate and severe stress experienced during the 12 months prior to the survey. Findings from previous studies reached similar conclusions regarding this association. For instance, based on a large sample of adult males, Cole and colleagues reported that perceived stress increased significantly among ‘common drinkers’ and problem drinkers compared to abstainers [20]. A longitudinal study found a positive effect only at the fourth follow-up (age 21) and only among men with relatively strong tension-reducing motivations for drinking [21]. In a study of more than 16,000 military personnel, stress was positively associated with heavy alcohol in the past month among men [22]. A study conducted in Canada, based on the CCHS 2010, reported that perceived stress increased the odds of infrequent Risky Single Occasion Drinking (RSOD) but not frequent RSOD [9].

It is important to note the possible role of alcohol intake as a coping mechanism to stress. That is, a higher stress level would lead to higher alcohol drinking [23, 24]. For instance, a recent report by the American College of Cardiology indicated that alcohol in moderation might help the heart by calming stress signals in the brain [25]. Alcohol abuse and dependence can often arise from the use of alcohol as a coping mechanism as it slows down the central nervous system, creating feelings of relaxation. Alcohol, as a depressant, is generally viewed as a coping mechanism for increased life stress arising from job insecurity and other life stressors [26]. On the reverse side, too much alcohol intake may sometimes lead to higher stress level as it could shift the hormonal balance and changes the way the body perceives stress and changes how it responds to stress [24, 27]. However, in the current study, the stress variables were measured in the past 12 months, while alcohol use was reported 7 days preceding the survey date. As a result, the stress variables are more likely to precede alcohol use, suggesting stress increases the risk of alcohol consumption.

A more interesting finding though was the interaction between racial background and the three key exposure variables discussed above (life stress, work stress and mental health). The expected mean of the WAC among the visible minority is much lower than for aboriginals and whites at all life stress levels. For work stress, the visible minority had a relatively stable and lower average alcohol intake compared to the other racial groups. On the other hand, Aboriginal respondents had steadily higher average alcohol intake for higher stress levels. A previous study reported that minority groups (blacks), especially those at the low end of the economic spectrum, report not only a great number of stressful life events but also stronger responses to them than whites in a variety of domains [28]. Another study reported that minority groups might suffer from both substance abuse and mental health disorders at high rates [29]. Some authors associate the role of race in alcohol abuse with differences in perceived cultural barriers, differences in economic status, differences in privilege, environmental and socio-cultural factors [29].

All socio-demographic characteristics considered in the present study appeared to have a strong and significant association with WAC. There was a significant sex difference in weekly alcohol consumption, where the expected mean number of WAC was higher for males compared to females. The finding is consistent with a recent Canadian study which reported that males were 4.69 times more likely to engage in risky alcohol consumption [9]. Marital status is another control variable predicting WAC in the present analysis.

Higher socio-economic status, as measured by educational attainment and income, was positively associated with alcohol consumption in our study. There was a non-linear relationship between income and WAC, with income group $20,000 to $39,999 having the lowest expected mean of WAC. A recent study in Canada reported that individuals in the highest income brackets had much higher odds of engaging in any RSOD, compared to those in the lowest income bracket controlling for all other factors, including education [9]. A study conducted in US, a country with a median household income of $67,521 in 2020 [30], reported that approximately78% of individuals with an income of $75,000 and above consumed alcohol, compared with 45% of those with an annual income of less than $30,000 [31]. Conversely, other studies indicated drinking alcohol is higher among low-income groups than higher incomes [10, 11]. Our study added to the existing literature and identified that higher income is associated with increased alcohol consumption. One plausible reason for the positive association between the two variables could be increased income usually boosts higher demand for alcohol [32].

A related variable, educational status, has shown a linear association where the expected mean weekly alcohol consumption was significantly higher for respondents with better education. Some previous studies reported that lower educational attainment was associated with increased risks of heavy drinking [33–35]. Nevertheless, a recent systematic review reported that people with higher socio-economic (income and education) might consume similar or greater amounts of alcohol compared with people with lower SES [36]. Similarly, another study on changes in alcohol consumption among adults (n = 6,787) concluded that persons with increasing consumption over time were more likely to be affluent and highly educated [32]. One possible explanation for the positive association between education and alcohol consumption could be the affordability of excessive alcohol or some people may use alcohol consumption as a vehicle for career advancement, especially at a younger age [37].

Finally, it is worth mentioning the strength and limitations of the study. Given that the findings were generated from a large sample, its implications for planning, monitoring, and evaluation of health programs are great. However, as the data were generated through a cross-sectional survey, causal inferences between two exposure variables and WAC were not possible. The self-report nature of WAC and the exposure variables might also impact the accuracy of the measurement. Finally, while inter-ethnic relations in people of aboriginal origin (or racism) may lead to higher alcohol consumption, CCHS did not collect such information, and hence, was not included in this analysis. The racial differentials are likely to contribute to alcohol consumption and create racial disparities in living conditions and overall health status [38, 39].

## Conclusion

The study concluded that the daily alcohol consumption is concerning as the mean number is high among the general adult population. It is further noted that the three main exposure variables (perceived work stress, perceived life stress, and perceived mental health) are significantly associated with the amount of weekly alcohol consumption, with their effects significantly differing across the racial groups. As the exposure variables are modifiable, the findings imply the need for continued public health education and behavioral change communication. Improving social support systems for individual/group counseling on the management of stressors and drivers could make significant program impacts. We recommend more studies investigating whether various job types interact with work stress. Also, future studies may pay more attention to whether certain preventive strategies (such as health promotion) impact the negative influence of stress on alcohol consumption.

## Data Availability

The dataset used for this study is made available by Statistics Canada: https://www.canada.ca/en/health-canada/services/food-nutrition/food-nutrition-surveillance/health-nutrition-surveys/canadian-community-health-survey-cchs.html.

## Abbreviations

**CCHS**: Canadian Community and Health Survey
**CI**: Confidence Interval
**RR**: Risk Ratio
**RSOD**: Risky Single Occasion Drinking
**SPSS**: Statistical Package for Social Sciences
**VIF**: Variable Inflation Factor
**WHO**: World Health Organization
